# Plant tolerance to high temperature in a changing environment: scientific fundamentals and production of heat stress-tolerant crops

**DOI:** 10.3389/fpls.2013.00273

**Published:** 2013-07-31

**Authors:** Craita E. Bita, Tom Gerats

**Affiliations:** Section Plant Sciences, Institute for Water and Wetland Research, Radboud University NijmegenNijmegen, Netherlands

**Keywords:** global warming, food security, heat tolerance, yield, productivity

## Abstract

Global warming is predicted to have a general negative effect on plant growth due to the damaging effect of high temperatures on plant development. The increasing threat of climatological extremes including very high temperatures might lead to catastrophic loss of crop productivity and result in wide spread famine. In this review, we assess the impact of global climate change on the agricultural crop production. There is a differential effect of climate change both in terms of geographic location and the crops that will likely show the most extreme reductions in yield as a result of expected extreme fluctuations in temperature and global warming in general. High temperature stress has a wide range of effects on plants in terms of physiology, biochemistry and gene regulation pathways. However, strategies exist to crop improvement for heat stress tolerance. In this review, we present recent advances of research on all these levels of investigation and focus on potential leads that may help to understand more fully the mechanisms that make plants tolerant or susceptible to heat stress. Finally, we review possible procedures and methods which could lead to the generation of new varieties with sustainable yield production, in a world likely to be challenged both by increasing population, higher average temperatures and larger temperature fluctuations.

## CLIMATE CHANGE UNDERCUTS GLOBAL FOOD SECURITY

Abiotic stresses are often interrelated, either individually or in combination, they cause morphological, physiological, biochemical, and molecular changes that adversely affect plant growth and productivity, and ultimately yield. Heat, drought, cold, and salinity are the major abiotic stresses that induce severe cellular damage in plant species, including crop plants. Fluctuations in temperature occur naturally during plant growth and reproduction. However, extreme variations during hot summers can damage the intermolecular interactions needed for proper growth, thus impairing plant development and fruit set. The increasing threat of climate change is already having a substantial impact on agricultural production worldwide as heat waves cause significantly yield losses with great risks for future global food security (Christensen and Christensen, 2007).

**FIGURE 1:**
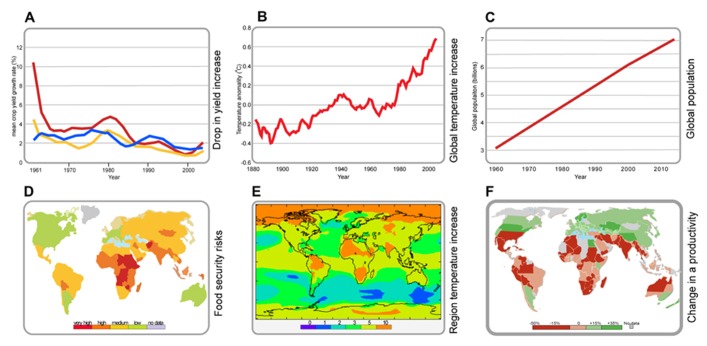
**Global temperature and population trends.** **(A)** Reduction in yield increases of major crops since 1961; wheat (red graph), rice (yellow graph), maize (blue graph). Data taken from FAO-STAT (). **(B)** Global increases in temperature since 1880. Data taken from Hansen et al. (2012). **(C)** Population increase since 1960. Data taken from the US Census Bureau (). **(D)** Regional food security risk areas. Data taken from  **(E)** Regional temperature increase. Data taken from the Hadely Centre, UK Met Office (). **(F)** Changes in regional agricultural productivity. Data taken from the Bard Center for Environmental Policy ().

Climatological extremes including very high temperatures are predicted to have a general negative effect on plant growth and development, leading to catastrophic loss of crop productivity and resulting in wide spread famine. Future agricultural production and thus global food security will encounter additional challenges from human population growth (**Figure 1**).

Presently, the Indian lowlands are the source of approximately 15% of global wheat production but it is anticipated that climate changes will transform these into a heat-stressed, short-season production environment. In a similar manner, a temperature increase of 3–4°C could cause crop yields to fall by 15–35% in Africa and Asia and by 25–35% in the Middle East (Ortiz et al., 2008). Based on a mathematical modeling, cereal production in Southeast Asia and Southern Africa is most likely to be affected by climate change if new strategies for amelioration are not found (Nelson, 2009; Fischer and Edmeades, 2010). Temperatures during the growing season in the tropics and subtropics may even exceed the most extreme seasonal temperatures observed till now, which will further aggravate the process of land degradation (Battisti and Naylor, 2009; Varshney et al., 2011). Latin America is predicted to experience increases in temperatures and decreases in precipitations throughout the continent but especially in Central America and the Caribbean. In Europe, most of the temperature increases will be in Southern and Central parts, the most affected countries being Spain, Portugal, and Italy. However, the changing climate conditions will potentially expand agricultural areas in the northern countries, where today crop cultivation is limited due to low temperature. In North America for example, the largest increase in temperature is expected at latitudes over 50°N, e.g., in Canada (Lotze-Campen and Schellnhuber, 2009).

Populations from developing countries are likely to be the most seriously affected as nearly 50% rely entirely on agriculture. In addition, 75% of the world’s poor live in rural areas. Thus, as population expands, crop production will have to be tailored to sustain food security and it has been suggested that world food production will have to increase by 70% to meet the demand of an expected population of 9 billion in 2050.

Despite the predicted increase in global food production, at present there is a major global food deficit and the relative rates of yield increase for the major cereal crops are declining (Easterling and Apps, 2005; Fischer and Edmeades, 2010; **Figure 1**). In many crop species, the effects of high temperature stress are more prominent on reproductive development than on vegetative growth and the sudden decline in yield with temperature is mainly associated with pollen infertility (Young et al., 2004; Zinn et al., 2010). Adding in the surging demand for food from the emerging economies such as China and India reveals an even more extreme challenge for plant breeders and farmers to such an extent that by 2050, the expected decline in calorie availability will aggravate malnutrition in children by 20% (Nelson, 2009; Chhetri and Chaudhary, 2011).

The growing food demand and the treat of heavy crop losses due to global climate change impose the urgent development of strategies to substantially improve food availability.

## MOLECULAR, CELLULAR, AND PHYSIOLOGICAL IMPACT OF HEAT STRESS IN PLANTS

Of the major forms of abiotic stress plants are exposed to in nature, heat stress has an independent mode of action on the physiology and metabolism of plant cells. Although frequently, heat stress is compounded by additional abiotic stresses such as drought and salt stress, it is important to unravel the independent action and biological consequences of high temperature in order to ameliorate the effects of combined abiotic stress. The susceptibility to high temperatures in plants varies with the stage of plant development, heat stress affecting to a certain extent all vegetative and reproductive stages. The observed effects depend on species and genotype, with abundant inter- and intra-specific variations (Barnabás et al., 2008; Sakata and Higashitani, 2008).

Various physiological injuries have been observed under elevated temperatures, such as scorching of leaves and stems, leaf abscission and senescence, shoot and root growth inhibition or fruit damage, which consequently lead to a decreased plant productivity (Vollenweider and Günthardt-Goerg, 2005). In many cases, plant architecture changes and hypocotyls and petioles elongate resembling the morphological responses of shade avoidance (Hua, 2009; Tian et al., 2009). However, high temperatures reduce plant growth by affecting the shoot net assimilation rates and thus the total dry weight of the plant (Wahid et al., 2007).

Higher plants exposed to excess heat, at least 5°C above their optimal growing conditions exhibit a characteristic set of cellular and metabolic responses required for the plants to survive under the high temperature conditions (Guy, 1999). These effects include changes in the organization of cellular structures, including organelles and the cytoskeleton, and membrane functions (Weis and Berry, 1988), accompanied by a decrease in the synthesis of normal proteins and the accelerated transcription and translation of heat shock proteins (HSPs; Bray et al., 2000), the production of phytohormones such as abscisic acid (ABA) and antioxidants and other protective molecules (Maestri et al., 2002).

**FIGURE 2:**
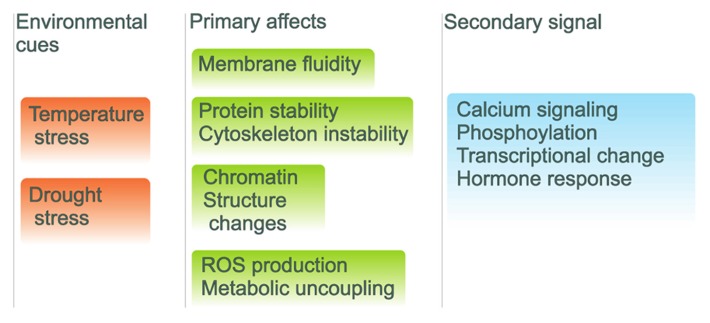
**Environmental signaling pathways with external cues and internal repercussions.** Environmental cues are on the left in red backgrounds with initial cellular effects with green backgrounds and the organismal signals are shown with blue background.

The changes in ambient temperature are sensed by plants with a complicated set of sensors positioned in various cellular compartments. The increased fluidity of the membrane leads to activation of lipid-based signaling cascades and to an increased Ca^2+^ influx and cytoskeletal reorganization. Signaling between these routes leads to the production of osmolytes and antioxidants in response to heat stress. For example, the *Arabidopsis *CNGC2 gene encodes a component of the membrane cyclic nucleotide gated Ca^2+^ channels that acts as the primary thermo-sensors of land plant cells. These channels in the plasma membrane respond to increments in the ambient temperature by triggering an optimal heat shock response (Saidi et al., 2009). This data emphasizes the vital role of lipid membranes in response to heat stress in plants (Horváth et al., 2012; **Figure 2**). Recently it was shown that differences in the tissue-specific activation of various signaling pathways may occur between vegetative and reproductive tissues (Mittler et al., 2011).

Heat stress induces changes in respiration and photosynthesis and thus leads to a shortened life cycle and diminished plant productivity (Barnabás et al., 2008). The early effects of thermal stress comprise of structural alterations in chloroplast protein complexes and reduced activity of enzymes (Ahmad et al., 2010). In addition, by causing injuries to the cell membrane, organization of microtubules and ultimately to the cytoskeleton, heat stress changes membrane permeability and alters cell differentiation, elongation, and expansion (Smertenko et al., 1997; Potters et al., 2008; Rasheed, 2009). For example, the photochemical modifications in the carbon flux of the chloroplast stroma and those of the thylakoid membrane system are considered the primary sites of heat injury (Wise et al., 2004), as photosynthesis and the enzymes of the Calvin–Benson cycle, including ribulose 1,5-bisphosphate carboxylase (Rubisco) and Rubisco activase are very sensitive to increased temperature and are severely inhibited even at low levels of heat stress (Maestri et al., 2002; Morales et al., 2003).

A specific effect of high temperatures on photosynthetic membranes includes the swelling of grana stacks and an aberrant stacking. Such structural changes are accompanied by ion-leakage from leaf cells exposed to heat and changes in energy allocation to the photosystems (Wahid and Shabbir, 2005; Allakhverdiev et al., 2008). The maintenance of cellular membrane function under high temperature stress is thus essential for a sustained photosynthetic and respiratory performance (Chen et al., 2010). The detrimental effects of heat on chlorophyll and the photosynthetic apparatus are also associated with the production of injurious reactive oxygen species (ROSs; Camejo et al., 2006; Guo et al., 2007). By increasing chlorophyllase activity and decreasing the amount of photosynthetic pigments, heat stress ultimately reduces the plant photosynthetic and respiratory activity (Todorov et al., 2003; Sharkey and Zhang, 2010). With respect to reproductive success, a decline of photosynthesis will eventually result in limited resource availability for reproduction in parental and gametophytic tissues due to a reduction in energy reserves leading to plant starvation (Young et al., 2004; Sumesh et al., 2008).

Homeostasis in general, including biosynthesis and compartmentalization of metabolites, is disturbed in high temperature challenged plant tissues (Maestri et al., 2002). High temperature modifies the activities of carbon metabolism enzymes, starch accumulation, and sucrose synthesis, by down-regulating specific genes in carbohydrate metabolism (Ruan et al., 2010). Among the primary metabolites accumulating in response to heat stress are proline, glycine betaine, or soluble sugars (Wahid, 2007). Many plant species accumulate other osmolytes as well, such as sugar alcohols (polyols), or tertiary and quaternary ammonium compounds (Sairam and Tyagi, 2004). Osmolyte production under heat stress is thought to increase protein stability and stabilize the structure of the membrane bilayer (Sung et al., 2003; Mirzaei et al., 2012).Secondary metabolites such as phenolics including flavonoids, anthocyanins, and plant steroids are also significantly involved in plant responses under heat stress and generally play roles in abiotic stress responses generally associated with tolerance to heat (Wahid, 2007). For example, thermal stress in tomato plants causes accumulation of soluble phenolics; increased phenylalanine ammonia-lyase activity; and decreased peroxidase and polyphenol oxidase activity, presumably as part of the acclimation to heat (Rivero et al., 2001).

Several key phytohormones including ABA, salicylic acid (SA), and ethylene (ET) also increase their levels under heat stress, while others decrease, such as cytokinin (CK), auxin (AUX), and gibberellic acids (GAs), fluctuations that ultimately cause premature plant senescence (Talanova et al., 2003; Larkindale and Huang, 2004; Larkindale et al., 2005). For example, the abscission of reproductive organs, an important effect of heat stress, is known to be caused by increased ABA and ET levels and reduced levels and transport of AUXs (Binder and Patterson, 2009). Similarly, an altered AUX biosynthesis in developing anthers was suggested to be related to pollen sterility (Sakata et al., 2010). Additionally, a comparable variation in CK content was also found to be the cause of reduced kernel filling in cereals (Banowetz et al., 1999).

Approximately 5% of the plant transcriptome is up-regulated twofold or more in response to heat stress in plants, and although often greatly induced by heat stress, chaperones are only a minor part of the general heat shock response (Larkindale and Vierling, 2008; Saidi et al., 2011). Most of the transcripts represent genes acting in primary and secondary metabolism, translation, transcription, regulation, and responses to environmental stresses, and in processes such as calcium and phytohormone signaling, sugar and lipid signaling, or protein phosphorylation.

Analogous to other abiotic stresses, heat stress results in the production of ROSs and invokes oxidative stress responses (Potters et al., 2007). Generating activated oxygen species under heat stress is a symptom of cellular damage, where membrane lipids and pigments peroxidation compromise membrane permeability and function. ROS cause damage to a wide range of cellular components such as the photosynthetic apparatus and various other components, hindering thus metabolic activities and affecting plant growth and yield by limiting metabolic flux activities (Sairam and Tyagi, 2004; Xu et al., 2006). The ROS related impairment of important mitochondrial and chloroplast electron transport chains associated with carbon metabolism results in reduced power and energy production (Foyer and Noctor, 2009). Although ROSs are clearly a direct cause of cellular damage on multiple levels, several studies have also shown that ROS play a key role as molecular signals, linking plant responses to pathogen infection, environmental stresses, programmed cell death (PCD), and even developmental stimuli (Gechev et al., 2006). ROS/redox signaling networks in the chloroplast and mitochondria have important roles in plant adaptation to abiotic stresses. These signals contribute to a complex interplay between organelles homeostasis under stress conditions and different cellular components and by controlling essential processes such as transcription, translation, energy metabolism, and protein phosphorylation (Mittler et al., 2011).

Subsequently, ROS production contributes to the transduction of the heat signal and expression of heat shock genes (Königshofer et al., 2008). Heat stress results in the misfolding of newly synthesized proteins and the denaturation of existing proteins. Protein thermostability is believed to be provided in part by chaperones, a specific class of proteins capable of assisting other proteins in proper post-translational folding and in maintaining them in a functional state (Ellis, 1990). In standard growth conditions the HSPs control cellular signaling, protein folding, translocation, and degradation, but under heat stress they prevent protein misfolding and aggregation and also act to protect cellular membranes. An increased production of HSPs occurs when plants experience either abrupt or gradual increases in temperature resulting in heat stress (Nover et al., 2001). There are considerable variations in the pattern of HSP gene expression in different species and even among genotypes within species. Heat shock factors (HSFs) are the transcriptional activators of HSPs (Banti et al., 2010). HSF regulation in *Arabidopsis* was shown to be positively regulated by small HSP and co-chaperones of the HSP90 complex such as ROF1, and negatively affected by an HSF binding protein (Meiri et al., 2010; Scharf et al., 2011). The HSP regulation may be achieved by a single “master switch” HSF or by the collective function of several HSFs, depending on the plant species (Baniwal et al., 2004; Liu et al., 2011).

A class of bHLH transcription factors known as phytochrome interacting factors (PIFs) has also been connected in the heat related signaling mechanisms. PIFs have a wide range of regulatory roles in photomorphogenesis, skotomorphogenesis, and the down-stream regulation of hormone levels, in particular AUX and GA and implicitly on other phytohormones such as ABA. The precise role of these proteins in heat stress remains unclear; however, the transcriptional response of PIF4 in particular has clear knock-on effects on early stages of plant development (Leivar and Quail, 2011).

Heat stress also leads to the transient activation of repetitive elements or silenced gene clusters close to the centromeric regions as well as the transient loss of epigenetic gene silencing (Lang-Mladek et al., 2010; Pecinka et al., 2010). Such gene silencing mechanisms are thought to be involved in transcriptional repression by hetero-chromatinization of repetitive DNA regions in plants (Khraiwesh et al., 2012). Recent studies have indicated that regulation of stress responsive genes often depends on chromatin remodeling. High temperatures cause transcriptional repression of genes involved in cell growth, such as histones and DNA polymerases and deregulation of DNA methylation and transposon activation (Sakata and Higashitani, 2008; Pecinka et al., 2010; Smith and Workman, 2012). For example, AtCHR12, a SNF2/Brahma (BRM)-type chromatin remodeling factor in *Arabidopsis* was shown to play a role in mediating the temporary growth arrest in response to drought and heat stress (Mlynárová et al., 2007). In addition, whereas histone modifications show only minor variations upon heat stress, there is evidence for a dramatic reduction in the number of nucleosomes associated with DNA, leading to loss of chromocenter organization, and this reduction in nucleosome density occurs throughout the genome. Efficient re-silencing of some of these activated targets seems to require the chromatin assembly factor 1 (CAF-1) complex (Larkindale et al., 2005). Further support for the important role of histone-mediated transcriptional regulation in the temperature response comes from the recent discovery of the histone H2A.Z variant, involved in the regulation of the temperature transcriptome in *Arabidopsis*. H2A.Z nucleosomes wrap DNA more tightly, which influences the ability of RNA polymerase (Pol) II to transcribe genes in response to temperature, suggesting a mechanism by which the transcriptome can be thermally regulated (Kumar and Wigge, 2010).

A wide range of plant developmental and physiological processes is negatively affected by heat stress. When the stress occurs at key developmental stages such as reproduction, this becomes one of the major constraints of plant adaptation to a changing environment (Hall, 2001). For example, high temperature during wheat reproductive development hastened the decline in photosynthesis and leaf area, decreased shoot and grain mass as well as weight and sugar content of kernels, while also reducing water-use efficiency (Shah and Paulsen, 2003).

Sexual reproduction and flowering in particular have been long recognized as extremely sensitive to heat stress, which often results in reduced crop plant productivity (Hedhly et al., 2009; Thakur et al., 2010). Studies carried out under glass and climate chambers suggest that high temperature is most deleterious at the stage of flower bud initiation, and that this sensitivity is maintained for 10–15 days (Hedhly et al., 2009; Nava et al., 2009). Many legumes and cereals show a high sensitivity to heat stress during flowering and severe reductions in fruit set have also been shown for several temperate and even tropical fruit trees species (Frank et al., 2009; Saha et al., 2010), most probably as result of reduced water and nutrient transport during reproductive development (Young et al., 2004).

The male gametophyte is particularly sensitive to high temperatures at all stages of development, while the pistil and the female gametophyte are considered to be more tolerant (Hedhly, 2011). Heat stress often accelerates rather than delays the onset of anthesis, which means that the reproductive phase of development will be initiated prior to the accumulation of sufficient resources (Zinn et al., 2010). The reproductive development in angiosperms takes place within two floral organs, the male stamen and the female pistil (Pachauri, 2007; Ahmad et al., 2010). The developmental pathway for the male gametophyte (the pollen grain) starts with the separation of the reproductive tissues of the anther, continues with meiosis of the pollen mother cell, followed by mitosis and microspore maturation that results in the mature pollen grain. After initiation, the highly specialized anther tissues will acquire non-reproductive (e.g., the tapetum for support, the stomium for dehiscence) or reproductive functions (the pollen mother cell for pollen formation). Both tapetum and microspore development are essential for male fertility, as documented by numerous studies on male sterile mutants (Prasad et al., 2003; Young et al., 2004; Christensen and Christensen, 2007; Tauber et al., 2007; Ainsworth et al., 2008; Jaggard et al., 2010; Zinn et al., 2010). Major variations in gene expression are observed under high temperature stress that are possibly linked to tapetum degeneration and pollen sterility in several plant species (Oshino et al., 2007; Endo et al., 2009).

Male sterility as a consequence of heat stress can be widely observed among many sensitive crop plants and the impairment of pollen development has been the main factor involved in reduced yield under heat stress (Sakata and Higashitani, 2008; Wassmann et al., 2009). For example, in barley and *Arabidopsis* anthers developing under high temperature (30–35°C), cell-proliferation is arrested, vacuoles are distended, chloroplast development is altered, and mitochondrial abnormalities occur (Sakata et al., 2010). Heat stress reduces carbohydrate accumulation in pollen grains and in the stigmatic tissue by altering assimilate partitioning and changing the balance between symplastic and apoplastic loading of the phloem (Taiz and Zeiger, 2006). Heat stress down-regulates sucrose synthase and several cell wall and vacuolar invertases in the developing pollen grains; as consequence, sucrose and starch turnover are disrupted and thus soluble carbohydrates accumulate in reduced levels (Sato et al., 2006). For example, in cowpea plants experiencing heat stress, drops in the concentration of soluble sugars in the anther walls, developing pollen grains, and in the locular fluid result in decreased sugar concentration in the mature pollen grains and decreased pollen viability (Ismail and Hall, 1999). In tomato, the reduction of sink- and source-strength even under moderately elevated temperatures leads to a depletion in available carbohydrates at critical stages of plant development, leading to reduced fruit set and other yield related parameters (Sato et al., 2006). In sorghum, heat stress reduces the accumulation of carbohydrate in pollen grains and ATP in the stigmatic tissue (Jain et al., 2007).

**FIGURE 3:**
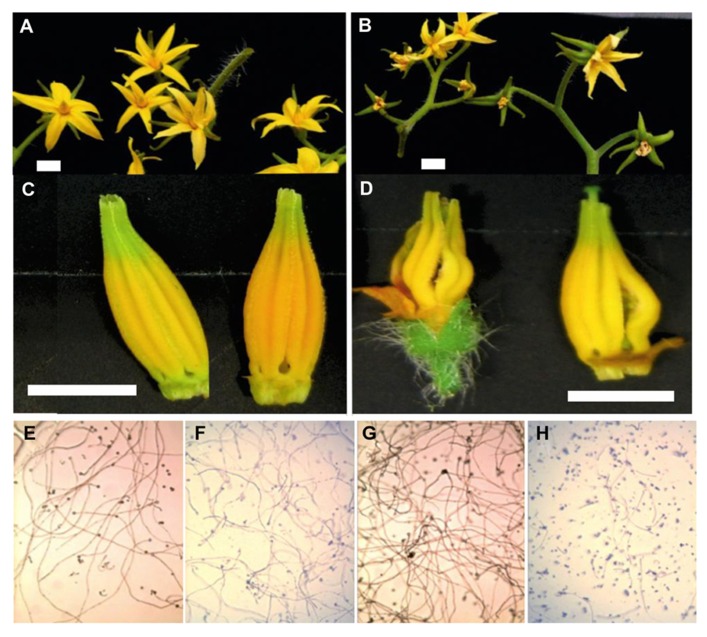
**Flower development and pollen viability of tolerant (left) and sensitive tomato genotypes (right).** The top shows inflorescences of a heat-tolerant genotype on the left **(A)** and a sensitive genotype on the right **(B)**. In the second panel, the morphology of the anther cones is shown for both genotypes **(C)** and **(D)**. Below **(E**–**H)** *in vitro* pollen germination under standard conditions (**E**; heat-tolerant genotype, **G**; heat-sensitive) and high temperature for both genotypes (**F**; heat-tolerant genotype, **H**; heat-sensitive).

Heat stress also induces early abortion of tapetal cells, which cause the pollen mother cells to rapidly progress toward meiotic prophase and finally undergo PCD, thus leading to pollen sterility (Oshino et al., 2007; Sakata and Higashitani, 2008; Parish et al., 2012). For example, under high temperature conditions, the structural abnormalities observed in developing microspores of snap bean anthers have been associated with tapetal degeneration due to malformations in the endoplasmic reticulum (Suzuki et al., 2001). Reduced dehiscence of tomato anthers under heat stress is also accompanied by closure of the locules and thus reduced pollen dispersal in several crop plants (Peet et al., 2002). We have also shown a number of such phenotypes in tomato flowers (**Figure 3**). We have shown differential effects of high temperature on the development of tomato flowers, anthers and the viability of tomato pollen in heat-sensitive and heat-tolerant lines.

Continuing heat stress beyond a successful fertilization can also halt further development of the embryo (Barnabás et al., 2008). Heat stress during seed development may result in reduced germination and loss of vigor, leading to reduced emergence and seedling establishment as has been shown for several crop plants (Akman, 2009; Ren et al., 2009). In many temperate cereal crops, both grain weight and grain number appear to be impacted by heat stress, with a decline in grain number directly proportional with increasing temperatures during flowering and grain filling (Porter and Semenov, 2005; Mahmood et al., 2010). Quality reductions in starch, protein, and total oil yield in several crop species have been also associated with heat stress during seed development (Banowetz et al., 1999). For example, high temperature during wheat reproductive development hastens the decline in photosynthesis and leaf area, decreases shoot and grain mass as well as weight and sugar content of kernels, and also reduces water-use efficiency (Shah and Paulsen, 2003). As a consequence, heat stress results in an altered nutritional flour quality (Hedhly et al., 2009).

In summary, high temperatures negatively affect various physiological processes including photosynthesis, primary and secondary metabolism, or lipid and hormonal signaling. Heat stress has negative effects on plant growth and development by disrupting the stability of various proteins, membranes, and cytoskeleton structures. The most affected stage is the reproductive growth and the affected process is pollen grain development. Heat induces accumulation of HSPs which prevent protein degradation and it also causes a state of metabolic imbalance and the build-up of toxic by-products, such as ROS, which ultimately affect plant vegetative and reproductive development, with negative consequences on fruit set and yield quality.

## PHYSIOLOGICAL AND MOLECULAR INDICATORS OF TOLERANCE TO INCREASED TEMPERATURES

Plants have evolved various mechanisms to ensure survival under elevated temperatures. These strategies are classified into long-term phenological and morphological evolutionary adaptations such as changing leaf orientation, transpirational cooling or alterations in the membrane lipid composition, or short-term stress avoidance and acclimation mechanisms. Among general stress tolerance mechanisms, stress proteins, osmo-protectants, free-radical scavengers, ion transporters and factors involved in signaling cascades and transcriptional control are essential to counteract stress effects (Wang et al., 2004).

### PHYSIOLOGICAL ASPECTS OF HEAT TOLERANCE

Producing an economically significant yield under heat stress conditions depends on several plant physiological parameters and mechanisms that contribute to heat tolerance in the field, such as amendments to essential processes such as photosynthesis, and concomitant increases of transcripts coding for proteins involved in protection. In many cases, a heat-tolerant variety is characterized by higher photosynthetic rates, increased membrane thermostability and heat avoidance (Nagarajan et al., 2010; Scafaro et al., 2010).

In all plant species, the ability to sustain leaf gas exchange under heat stress is directly correlated with heat tolerance. As outlined above, the decrease in the amount of active Rubisco can account for a large proportion of the negative temperature response of net photosynthesis (Salvucci and Crafts-Brandner, 2004) and the primary target site of thermal injury is carbon fixation by Rubisco. The reduction in carbon fixation and the consequent oxygen evolution result in generation of harmful ROS. As a consequence, the repair mechanism of the damaged photosystem is inhibited. Hence, approaches to develop crops with improved productivity in a high temperature environment could include manipulating leaf photosynthesis and even photosynthate partitioning (Ainsworth and Ort, 2010).

One of the typical heat stress symptoms is tissue senescence, characterized by membrane damage associated with increased fluidity of membrane lipids, lipid peroxidation, and protein degradation in various metabolic processes (Savchenko et al., 2002). Membrane lipid saturation is therefore considered an important element in high temperature tolerance. A higher share of saturated fatty acids in membrane lipids increases the lipid melting temperature and prevents a heat-induced increase in the membrane fluidity. Therefore, to maintain membrane fluidity, plants increase the content of saturated and monounsaturated fatty acids, modulating their metabolism in response to increasing temperatures (Zhang et al., 2005b). Thus, increasing the saturation level of fatty acids appears to be critical for maintaining membrane stability and enhancing heat tolerance (Larkindale and Huang, 2004).

Accumulation of osmo-protectants is an important adaptive mechanism in plants subjected to extreme temperatures, as primary metabolites participate directly in the osmotic adjustment (Sakamoto and Murata, 2000). For instance, accumulation of proline, glycine betaine, and soluble sugars is necessary to regulate osmotic activities and protect cellular structures from increased temperatures by maintaining the cell water balance, membrane stability, and by buffering the cellular redox potential (Farooq et al., 2008). Transgenic approaches have confirmed the beneficial effect of proline overproduction during stress, as an enhanced proline production in transformed plants correlates well with a more negative leaf osmotic potential and higher production of protective xanthophyllic pigments under heat (Dobra et al., 2010).

Glycine betaine plays an important role as compatible solute in plants experiencing high temperature conditions (Sakamoto and Murata, 2002). Glycine betaine production in chloroplasts maintains the activation of Rubisco by sequestering Rubisco activase near thylakoids and preventing its thermal inactivation (Allakhverdiev et al., 2008). For example, high levels of glycine betaine accumulation were reported in maize and sugarcane in response to high temperature, while in contrast, plant species such as rice, mustard, *Arabidopsis*, and tobacco naturally do not produce glycine betaine under stress conditions (Quan et al., 2004; Wahid and Close, 2007).

Studies suggest that high carbohydrate availability (e.g., glucose and sucrose) during heat stress represents an important physiological trait associated with heat stress tolerance (Liu and Huang, 2000). Sucrose is the principal end product of photosynthesis, which translocates from source leaves to sink organs through the phloem. Sucrose and its cleavage products regulate plant development and response to stresses through carbon allocation and sugar signaling (Roitsch and González, 2004). Studies on an a heat-tolerant tomato genotype demonstrated that it is the high cell wall and vacuolar invertases activities and increased sucrose import into young tomato fruit that contribute to heat tolerance through increasing sink strength and sugar signaling activities (Li et al., 2012). Similarly, the carbohydrate content of developing and mature pollen grains may be an important factor in determining pollen quality, as heat-tolerant tomato cultivars appear to have a mechanism for maintaining the appropriate carbohydrate content under heat stress (Firon et al., 2006). In addition, sugars have been shown to also act as antioxidants in plants (Lang-Mladek et al., 2010). At low concentrations sucrose acts as signaling molecule while it has been suggested that in high concentrations it becomes a ROS scavenger (Sugio et al., 2009).

Enhanced synthesis of secondary metabolites under heat stress conditions also protects against oxidative damage. Several studies in tomato and watermelon indicate that thermal stress induces the accumulation of phenolics in the plant by activating their biosynthesis as well as inhibiting their oxidation, which could be an acclimation mechanism of the plant against thermal stress (Rivero et al., 2001). It has been suggested that in addition to their role as UV screen, anthocyanin accumulation under heat stress serve to decrease leaf osmotic potential, resulting in an increased uptake and reduced transpirational loss of water. These properties may enable the leaves to respond quickly to changing environmental conditions (Wahid et al., 2007).

Carotenoids protect various plant species from several stresses. For example, xanthophylls and some other terpenoids such as isoprene or tocopherol stabilize and photo-protect the lipid phase of the thylakoid membranes (Velikova et al., 2005; Camejo et al., 2006). Similarly, *Arabidopsis* plants overexpressing the chyB gene that encodes b-carotene hydroxylase (an enzyme active in the zeaxanthin biosynthetic pathway) show a greater tolerance to increased temperatures, and it was suggested that the protection from stress is most likely due to the action of zeaxanthin in preventing oxidative damage to membranes (Meiri et al., 2010).

Several lines of evidence indicate that separate from HSP induction, other pathways are involved in acquiring tolerance to heat. Several plant growth regulators, such as ABA, SA, ET, CK, and AUX are proposed to play an important role in plant thermotolerance (Kotak et al., 2007). Under field conditions, ABA induction is an important component of thermotolerance due to its implication in survival under heat and desiccation stress (Maestri et al., 2002) and it is generally accepted that the ability to synthesize ABA under heat stress is the key factor attributed to the higher heat tolerance of plant cells (Ding et al., 2010). It has also been noted that the plant hormone ABA induces thermotolerance in maize (Musatenko et al., 2003). Pretreatment with SA increases basal tolerance to heat in an *Arabidopsis* mutant defective in SA signaling. In addition, transgenic plants unable to accumulate SA show up to 40% reduction in tolerance to heat (Kaya et al., 2001; Clarke et al., 2004). The maintenance of high levels of CKs in the kernels during heat stress appears to be important in increasing thermotolerance and providing yield stability in crop systems, as it is known that CKs have the potential to reduce oxidative stress in plants (Hare et al., 1997; Hsu et al., 2010). Foliar application of seaweed extract-based CK has been shown to increase leaf CK content and delay senescence of *Agrostis* sp. under heat and drought stress (Zhang and Ervin, 2008). In barley as well as in *Arabidopsis*, heat stress represses AUX signaling in an anther-specific manner, leading to abortion of pollen development. As applying exogenous AUX entirely restores pollen development in heat conditions, it has been postulated that AUX promotes fertility under heat stress (Jaggard et al., 2010). In tomato and *Arabidopsis*, brassinosteroids also confer tolerance to high temperature stress by inducing the biosynthesis of major HSPs (Ogweno et al., 2008; Bajguz and Hayat, 2009). Similarly, epibrassinolide treatment modulates the translational machinery, resulting in higher HSPs synthesis and rapid resumption of protein synthesis during and following the application of high temperature stress in *Arabidopsis* and rapeseed (Kagale et al., 2007).

### MOLECULAR ASPECTS OF HEAT TOLERANCE

Plants are capable of adapting to a wide range of temperatures by reprogramming their transcriptome, proteome, and metabolome and even by activating cell death mechanisms leading to organ abortion or entire plant death (Qi et al., 2011; Sánchez-Rodríguez et al., 2011). The ability to withstand or to acclimate to higher than optimal temperatures results from repair of heat-sensitive components and prevention of further heat injury, metabolic homeostasis being also maintained during stress. The most important characteristic of thermotolerance is the massive production of HSPs (Vierling, 1991), however, as heat tolerance is a multigenic character, numerous biochemical and metabolic traits are also involved in the development and maintenance of thermotolerance: antioxidant activity, membrane lipid unsaturation, gene expression and translation, protein stability, and accumulation of compatible solutes (Kaya et al., 2001). Nevertheless, plant responses to high temperatures clearly depend on genotypic parameters, as certain genotypes are more tolerant (Prasad et al., 2006; Challinor et al., 2007).

Several studies revealed that while some HSFs are critical for thermotolerance, others play a less critical role (HSP101, HSA32, HSFA1, HSFA3), since knockout variants of these proved to have little impact on tolerance to heat (Larkindale and Vierling, 2008; Schramm et al., 2008; Yoshida et al., 2011). These results also indicate that a complex regulatory network delivers a differential protection from heat stress. A recent study shows that in the absence of the HSFA1 transcription factor, a minimal yet significant level of acquired thermotolerance can still be attained in *Arabidopsis* mutants following acclimation, likely due to the induction of a small number of genes regulated by other transcription factors such as bZIP28 (Liu and Charng, 2012). Nevertheless, HSPs are of particular importance in thermotolerance reactions and act as molecular chaperones to prevent denaturation or aggregation of target proteins as well as facilitating protein refolding (Lohar and Peat, 1998; Ahuja et al., 2010; Scharf et al., 2011).

In many plant species, thermotolerance of cells and tissues after a heat stress is pretty much dependent upon induction of HSP70, though HSP101 has also been shown to be essential (Gurley, 2000). The enhanced expression of HSP70 was reported to assist in translocation, proteolysis, translation, folding, aggregation, and refolding of denatured proteins (Gorantla et al., 2007; Zhang et al., 2010), while a methionine-rich chloroplast HSP has been shown to protect the thermolabile photosystem II and, consequently the whole-chain electron transport during heat stress (Peet et al., 2002). HSP101 for example, appears not to be required for growth in normal conditions, but plays a major role in tolerance to severe heat stresses and protein oxidative protection in *Arabidopsis *(Queitsch et al., 2000). Recently, it was, however, shown that an *Arabidopsis* mitochondrial transcription termination-factor related protein enhances thermotolerance in the absence of HSP101, via mitochondrial oxidative damage control (Kim et al., 2012). Several studies support the hypothesis that HSFs can function as molecular sensors that are able to directly sense ROSs such as H_2_O_2_ and control the expression of oxidative stress response genes during oxidative stress in *Arabidopsis* and tomato (Miller and Mittler, 2006; Volkov et al., 2006). Of the HSP gene family, induction of small HSP gene expression and protein accumulation upon environmental stresses point to the hypothesis that these proteins play an important role in stress tolerance (Sun et al., 2002). For example, some sHSPs become membrane associated, forming heat shock lipids that can stabilize membranes early on during the thermal stress. Introduction of the sHSP17.7 gene from carrot to potato was shown to enhance thermotolerance by affecting cellular membrane stability (Hu et al., 2010).

Recent studies also implicate the chloroplast protein synthesis elongation factor (EF-Tu) in plant response to high temperature stress. EF-Tu displays chaperone activity (Fu et al., 2008), and it has been suggested that high temperature-induced accumulation of EF-Tu is of importance in plant tolerance to high temperature stress (Fu et al., 2008; Prasad et al., 2008), as cultivars expressing a higher EF-Tu under high temperature stress were more tolerant to high temperature stress (Pressman et al., 2002).

*In vitro* experiments from several laboratories suggest that thermal stress at relevantly high temperatures produces ROS such as superoxide radicals, hydroxyl radicals, and hydrogen peroxide at the chloroplastic PS II reaction center, which are scavenged by antioxidants, including superoxide dismutase (SOD; Bukhov and Mohanty, 1999). Tolerant plants generally protect themselves from the damaging effects of ROS with the synthesis of various antioxidant components, which control gene expression and influence essential processes such as growth, PCD, abiotic stress responses, and pathogen defense (Abiko et al., 2005). These components have been found in almost all cellular compartments, indicating the importance of ROS detoxification for cellular survival (Iba, 2002; Mittler et al., 2004; Asada, 2006). Essential for ROS detoxification particularly during stress are antioxidants such as ascorbic acid or glutathione and ROS-scavenging enzymes such as SOD, ascorbate peroxidase (APX), catalase (CAT), or glutathione peroxidase (GPX).

In *Arabidopsis* for example, the APX gene family expression is heat stress dependent and regulated by HSF, evidence that links the heat stress response with oxidative stress and stress tolerance (Panchuk et al., 2002). Recently it was reported though that not all family members are positive regulators of tolerance to heat, as *Arabidopsis* plants deficient in an APX gene show enhanced seed production under extended heat stress conditions (Suzuki et al., 2013). Amelioration of oxidative damage to membranes could also represent another mechanism for maintaining membrane stability under heat stress (Kotak et al., 2007) and it has been recommended that targeting detoxification pathways might be an appropriate approach to engineer plants with multiple stress-tolerance traits (Ristic et al., 2009).

Real time measurements of cytosolic calcium levels during heat stress in *Arabidopsis* show transient elevations in response to recovery from heating. The magnitude of these calcium peaks is greater in thermo-tolerant plants, implying that these calcium signals might play a role in mediating the effects of thermotolerance. Thus, examination of Ca^2+^ flux may allow the selection of heat-resistant varieties. Moreover, identification of the key players may allow targeting of the heat shock response to particularly relevant tissues, such as reproductive structures (Mach, 2012).

In plants, the acetylcholine (Ach)-mediated system, composed of ACh, ACh receptor (AChR), and AChE plays a significant role in signal transduction, as it does in animals. Overexpression of maize catabolic gene AChE in transgenic tobacco plants enhances heat tolerance relative to that of non-transgenic plants, suggesting thus that AChE plays a positive role not only in maize but also in tobacco heat tolerance. Native tropical zone plants also show high AChE activity during heat stress. Therefore, engineering plant AChE might be useful in breeding for enhanced heat tolerance (Yamamoto et al., 2011). Heat-induced expression of various proteases is also important in regulating plant responses to heat stress. For example, the FtsH11 protease of *Arabidopsis* has been found to contribute to overall tolerance to high temperatures by alleviating light stress through the degradation of unassembled thylakoid membrane proteins (Chen et al., 2006). HOT5, which encodes an alcohol dehydrogenase functioning as nitrosoglutathione reductase, is also required for survival under heat, revealing a possible role of nitric oxide (NO) in thermotolerance and plant development (Sivasankar et al., 2012).

Late embryogenesis abundant (LEA) proteins, ubiquitin, and dehydrins have been found to play important roles in protection from heat and drought stress. For example, LEA proteins can prevent aggregation and protect the citrate synthase (involved in ATP production) from desiccating conditions like heat and drought stress (Willits and Peet, 1998). Similarly, ubiquitin and conjugated-ubiquitin synthesis during the first 30 min of exposure emerged as an important mechanism of heat tolerance in mesquite and soybean experiencing heat stress (Huang and Xu, 2008).

With respect to tolerance responses of reproductive tissues, approaches to discover the molecular mechanisms conferring heat tolerance during pollen development are a valuable avenue help develop heat-tolerant germplasm. Genome-wide strategies are being used to investigate all aspects of pollen development, including responses to temperature stress (Frank et al., 2009; Jagadish et al., 2010; Bita et al., 2011). Several studies reveal that HSPs are not the only players involved in response to heat in the reproductive structures, but other components are involved as well, such as hormones and antioxidants (Sakata et al., 2010).

Several genes have been identified in tolerant genotypes or wild germplasm, which were shown to improve photosynthesis and protect reproductive development, such as HSP101, stable Rubisco isoforms, etc. However, many genes identified from anther profiling under heat show no homology to known sequences and represent potential candidates for further study of the tolerance to heat (Zinn et al., 2010). An analysis of tomato maturing microspores revealed increased expression of SlAPX3 (a ROS scavenger), ET responsive genes (including the MBF1 homolog ER24), HSFA2, and HSFA3, and HSP family members. In addition, a calcium dependent protein kinase 2 (CDPK2) was up-regulated in the heat-stressed microspores, but whether the heat stress induction of this gene results in a more active stress tolerance response is still to be determined (Frank et al., 2009).

High temperature stress is detrimental to cereal crop productivity and the existence of genetic variability in heat stress tolerance is an indispensable factor for the development of more tolerant cultivars. For example, tolerant wheat genotypes are defined by maintenance of photosynthesis, chlorophyll content, and stomatal conductance under heat stress, while the yield of these genotypes is maintained through higher seed set, grain weight, and extended grain filling duration (GFD) even at elevated temperatures (Yang et al., 2002). When there is a limited variability for tolerance to heat within a crop species, wild germplasm can also be used as tolerance source (Pradhan et al., 2012b).

In a heat-tolerant rice variety, multiple protein components of the Calvin cycle increased in expression under heat stress, including a consistent increase of phosphoribulokinase (PRK), the enzyme responsible for the final step in ribulose-1,5-bisphosphate (RuBP) regeneration. The protective proteins Cpn60, HSP90, and HSP70 also increased not only in protein abundance but also in gene expression and even a thiamine biosynthesis protein (THI1), previously shown to have a protective role against stress, increased its levels during the heat stress (Scafaro et al., 2010).

Similarly, a proteomic analysis comparing proteins expressed in heat-stressed anthers from three rice varieties with different temperature tolerances, revealed a higher accumulation of sHSPs in the most tolerant rice genotype, compared with the most sensitive rice genotype, while the moderately tolerant rice genotype showed intermediate sHSPs accumulation. It was therefore proposed that the accumulation of sHSPs might confer greater heat tolerance in rice (Jagadish et al., 2010).

Genetic variation in the ability of tomatoes to set fruit under high temperature conditions, has made selection for heat tolerance possible. For example, in several genotypes differing in their capacity for thermotolerance, as well as in sugarcane, an increased chlorophyll a:b ratio is observed in the tolerant genotypes under high temperatures, indicating that these changes are related to thermotolerance (Camejo et al., 2006; Wahid, 2007). A comparative anther transcriptome analysis between tolerant and sensitive tomato genotypes shows that the metabolism and development-related genes are more highly expressed in the heat-sensitive genotypes, while gene modulation intensity suggests that the difference in tolerance to heat stress is associated with a lower percentage of transcripts affected in the heat-tolerant genotypes; however, HSP induction remains higher in the tolerant genotypes (Frank et al., 2009; Bita et al., 2011). Several tolerant grape genotypes show higher levels of HSP70 and genes related to metabolism and stress protection under elevated temperatures and one tolerant genotype showed higher heat shock gene expression levels even under standard conditions (Zhang et al., 2005a).

White goosefoot populations native to more stressful habitats or grown at higher temperatures have lower HSP levels and induced thermotolerance, relying apparently on basal mechanisms for thermotolerance and it has been suggested that future global climate change will differentially impact ecotypes within species, possibly by selecting for increased basal versus inducible thermotolerance (Barua et al., 2008). Indeed, higher basal gene expression levels under non-challenged conditions have also been measured for microspores of a heat-tolerant tomato cultivar. The detected genes are considered as interesting candidates for microspore thermotolerance (Frank et al., 2009). Hence, the inherent genetic variability for tolerance can be exploited to our advantage and genes that confer stress tolerance can be sourced from germplasm collections, wild relatives or organisms that live in extreme habitats.

Global warming will have significant impacts on future crop yields. In the view of the predicted population growth and the resulting increasing requirement for food security, it is up to the scientific community to adapt crop species for high tolerance to abiotic stresses and in particular high temperature stress. A more complete insight of the biological processes behind the heat stress response combined with classical and emerging technologies in plant breeding and genetic engineering is likely to make a significant contribution to improved crops.

## STRATEGIES TO CROP IMPROVEMENT FOR HEAT STRESS TOLERANCE

Generally, tolerance to heat is characterized by a lesser effect on essential processes such as photosynthesis and by consistent increases of transcripts involved in the biosynthesis of protective components. As photosynthesis and reproductive development are the most sensitive physiological processes to stress (Prasad et al., 2008), a heat-tolerant variety will be usually characterized by higher photosynthetic rates reflected in stay-green leaves, increased membrane-thermostability and successful fruit set under high temperature conditions (Nagarajan et al., 2010; Scafaro et al., 2010).

Multiple opportunities for plant improvement exist, as tolerance to high temperatures is a multigenic character. Screening for heat tolerance in the field presents a challenge due to interactions with other environmental factors but a wide variety of screenable traits is available that allows successful selection in the field (Hall, 2011). Tolerant genotypes may also be selected in controlled environments. However, besides being more expensive, controlled environments do not allow natural selection for other factors that interact with the heat stress tolerance mechanisms under field conditions (Souza et al., 2012). Regardless the screening method, a key objective for plant breeders is to develop an effective set of thermotolerance markers which can be used to further implement heat tolerance into various crop species.

Since plants adapt to temperature stress by developing more appropriate morphological, physiological, and biochemical characteristics, analyzing plant phenology in response to heat stress often gives a better understanding of the plant response and facilitates further molecular characterization of the tolerance traits (Wahid et al., 2007). The emerging phenomics methodologies are used to identify genes associated with traits of interest by establishing functional relationships between genetics and the associated phenotype. Such tools are also used to characterize plant performance under controlled environments or in the field (Sivasankar et al., 2012). The temperature and duration of heat stress treatments resulting in changes in growth and development vary between plant tissues and growth stages so choosing an appropriate phenotype is critical as the function of a heat stress response gene may contribute to thermotolerance differentially across tissues and growth stages. Ideally, systematic phenotyping approach that includes a range of heat stress conditions may increase the chances of identifying the functions of potential heat stress response genes.

Despite previous characterization of genes that ameliorate the effects of high temperature, direct trait selection based on molecular markers remains difficult. Thus, a more productive alternative to genetic engineering would be selection for indirect physiological traits related to tolerance. For example, in many crop plants, early maturation under high temperatures is closely correlated with smaller yield losses (Adams et al., 2001). Furthermore, the genetic variability for tolerance to heat stress present in rice could be exploited to screen germplasm and select cultivars that open flowers earlier in the morning or that maintain a high number of spikelets/panicle when grown in warm environments (Singh et al., 2010). Similarly, in wheat, a positive correlation between canopy temperature depression (CTD) and grain yield has been reported and recommended as a useful trait in selecting high temperature tolerant genotypes (Pradhan et al., 2012a).

At cellular level, electron transport rate, membrane integrity, and enzyme viability have also been successfully used to screen for heat tolerance under field conditions (Cottee et al., 2010). Chlorophyll accumulation assays have been used to characterize genetic variability in acquired thermotolerance for many crop species (Selvaraj et al., 2011). In wheat for instance, heat stress-induced damage of the thylakoid membrane is closely associated to chlorophyll loss, and detection of chlorophyll content has been proposed as high throughput screening method for tolerance to heat (Shah and Paulsen, 2003).

Pollen viability and seed-set as measures of reproductive success under heat stress are also candidate traits for selection in breeding programs, and generally there is a strong correlation between pollen production and viability, anther dehiscence, and seed-set. The anthers of heat-tolerant rice cultivars dehisce more easily than those of susceptible cultivars under high temperature conditions (Prasad et al., 2006; Jagadish et al., 2010).

Breeding for stress tolerance requires efficient screening procedures, identification of key traits in diverse donor or tolerant lines and understanding their inheritance and molecular genetics. Molecular genetic markers are an example of how an effective tool is used to analyze plant genomes and how heritable traits associate to their underlying genetic variation. Sequence-based (microarrays) or anonymous molecular marker systems [amplified fragment length polymorphism (AFLP); Vos et al., 1995] are often employed in applications of modern plant genetic analysis. Moreover, the reducing cost of DNA sequencing and increasing availability of large sequence data sets will allow mining for large numbers of such markers. Further on, the markers can be introduced in genetic linkage analyses and trait mapping, association studies and marker-assisted selection (MAS; Duran et al., 2010). At this time, sequencing technology is no longer the bottleneck for the generation of sequence data from whole-genome shotgun sequencing (WGS) and thus, the generation of marker data has become a bioinformatics issue rather than a cost or time issue (Egan et al., 2012). For the major crops, including those that could deliver the largest impact on improvement of yield security under the expected higher global temperatures, re-sequencing will become the method of choice for identifying markers that co-segregate with temperature tolerance traits. The wealth of marker data generated by WGS, facilitates the implementation of these very high resolution marker datasets for quantitative trait locus (QTL) mapping and genome-wide association studies (GWAS).

The search for molecular markers associated to phenotypic traits is one aspect of molecular genetics usually carried out with methods based on segregation mapping, genomic introgression, and association mapping (Morgante and Salamini, 2003). For example, QTL mapping has recently become the method of choice to identify specific chromosome segments that contain candidate genes for heat tolerance (Argyris et al., 2011; Zhang et al., 2012).

Quantitative trait locus analysis in tolerant and sensitive crops is now receiving much attention. The key benefit of QTL-based approaches is that they allow loci to be identified that are linked to heat tolerance. The identification of markers linked to QTLs enables breeding of stress-tolerant crops by combining or “pyramiding” QTLs for tolerance to various stresses. Several QTL studies relating to various abiotic stress tolerances have already been reported (Hirayama and Shinozaki, 2010).

Detecting adaptive QTLs for high temperature tolerance is one way to understand tolerance mechanisms and several studies have already identified genetic markers related to different environmental stresses, including heat (Roy et al., 2011). QTL mapping studies for heat tolerance have been conducted on various rice populations at flowering stages. However, confirmation and fine mapping of the identified QTLs for heat tolerance have not been reported yet (Ye et al., 2012). Multiple loci for heat tolerance have been identified in wheat (Paliwal et al., 2012) and maize (Bai, 2011). A study on *Arabidopsis* mutants sensitive to heat also revealed QTLs involved in acquiring thermotolerance (Hong et al., 2003). A major QTL for high temperature germination and an additional QTL having smaller effects were identified as well in a genetic analysis of lettuce seed thermo-inhibition (Argyris et al., 2008). The markers linked to these QTLs could be used to improve heat tolerance in available germplasm. At present, high temperature tolerance QTL identification is performed using different traits, such as the thousand-grain weight (TGW), the GFD, CTD, yield (Pinto et al., 2010), or senescence related traits (Vijayalakshmi et al., 2010).

In order to transfer these traits, classical breeding requires the establishment of rapid and cost effective screening procedures and implementing these using conventional non-transgenic breeding approaches such as “marker-assisted screening,” association mapping or genomic selection (GS) procedures.

Recently, association genetics has started to assist in QTL identification in several crop species (Ahuja et al., 2010). Association mapping or linkage disequilibrium mapping is a high resolution and relatively low cost methodology, that might likely be used to identify traits associated with abiotic stresses, in combination with high throughput marker genotyping platforms (Varshney et al., 2009). Once the markers associated with QTLs have been isolated, the candidate QTLs can be further introgressed in elite lines through MAS strategies. One of the difficulties of developing superior genotypes for heat stress is that these traits are generally controlled by small effect QTLs or several epistatic QTLs. In order to overcome this problem, pyramiding several QTLs from large populations in the same genetic background, marker-assisted recurrent selection (MARS) or GS approaches can be engaged (Tester and Langridge, 2010).

Marker-assisted selection approaches have significantly contributed to revealing the genetic basis of plant stress tolerance in some crops and even led to the release of plants with enhanced tolerance to abiotic stress (Lopes and Reynolds, 2010; Thomson et al., 2010). However, MAS programs for complex traits such as tolerance to heat are not effective mainly due to the genotype × environment and gene–gene (i.e., epistasis) interactions, which frequently results in a low breeding efficiency (Collins et al., 2008). In contrast to MAS strategies which use markers for which a significant association with a trait has been identified, the GS method predicts breeding values using data derived from a vast number of molecular markers with a high coverage of the genome. Its novelty is that it uses all marker data as predictors of performance and subsequently delivers more accurate predictions. Simulation studies indicated that GS may increase the correlations between predicted and true breeding value over several generations, without the need to re-phenotype. Thus, GS may result in lower analysis costs and increased rates of genetic gain (Habier et al., 2009; Heffner et al., 2009).

However, QTLs often do not translate well across genetic backgrounds and often produce smaller than expected adaptation effects. To improve crop abiotic stress tolerance by exploiting the segregation of natural alleles seems thus rather challenging for such an adaptive QTL strategy (Collins et al., 2008). When quantitative hereditary characteristics such as heat stress tolerance are involved, recurrent selection seems to be one of the most efficient methods in plant breeding. In multiple crosses, the probability is very small of obtaining superior genotypes that reunite all the favorable alleles. However, in this circumstance, a large segregating population is required, aspect that becomes unfeasible in practice. The alternative is to adopt recurrent selection to gradually accumulate, by recombination cycles, the desirable and available alleles in different parents (Doná et al., 2012). The primary purpose of a recurrent selection program is to increase the frequency of favorable alleles for traits of interest, conserving the genetic variability. Advantages are (a) greater genetic variability obtained by inter-crossing of multiple parents; (b) greater opportunity for recombinations because of successive crossings; (c) greater efficiency in increasing the favorable gene frequency because the process is repetitive and accumulative; (d) greater facility to incorporate exotic germplasm in the population (Ramalho et al., 2005). For example, the potato breeding program at the Federal University of Lavras has also been successful in developing heat stress tolerance genotypes using recurrent selection obtaining expressive gains in tuber production with improved quality (Benites and Pinto, 2011).

Obviously, only field trials under real stress conditions allow for conclusive remarks on stress tolerance and yield performance of a genotype (Mason et al., 2010). However, the lack of a precise phenotyping protocol is most probably the limiting factor in the genetic analysis of quantitative traits. Development of more precise phenotyping tools that can be applied to field conditions is a prerequisite for enabling the assessment of the complex genetic networks associated with QTLs. The small yet significant phenotypic changes delivered by introducing single genes into breeding material require precision phenotyping protocols and the capacity to carry these out on very large populations (Cattivelli et al., 2008).

To transfer advantageous traits to a cultivar, modern (genetic modification) approaches can also be employed. The transgenic approach requires identification of the gene responsible for the desired trait but poses no barrier to transferring useful genes across different species within the plant kingdom or even from animal systems. Genes of non-plant species could potentially be introgressed as well and generally, several combinations of beneficial genes could be transferred into the same plant. Transformation protocols are available for most important food crop species. However, the regulatory restrictions for the use of transgenic plants make this technology economically unpractical. Moreover, the existing methods have a diminished efficiency for cereal crops such as wheat and barley and even rice (Takeda and Matsuoka, 2008). Nevertheless, to combine stress tolerance with high yield potential while avoiding the negative effects of a stress gene on plant growth under favorable conditions, strategies that spatially and temporally restrict transgene expression via tissue-specific and stress-inducible promoters are used (Nakashima et al., 2007). Engineering promoters will facilitate gene pyramiding through genetic modification, addressing the issue of tolerance to multiple stresses at different stages of plant growth (Datta et al., 2002). As an alternative, engineering with specific transcription factors and signaling components could be employed. Ultimately that leads to the expression of their target transcriptome that consists of several genes involved in the response to stress. Transcriptome engineering emerges thus as a promising avenue for the development of abiotic stress-tolerant crops. Currently, however, plant genetic engineering is hampered by non-biological constraints mainly related to the commercialization of transgenic crops, particularly in Europe (Cattivelli et al., 2008). Thus, the future commercial success of transgenic breeding will depend upon the development of clearly defined and scientifically based regulatory frameworks, and upon public acceptance of genetically modified plants and their produce (Godfray et al., 2010).

## CONCLUSION

It is now well accepted that the complexity of the heat syndrome can only be tackled with a holistic approach that integrates examination of crop heat tolerance traits by classical and modern molecular genetic tools with agronomic practices resulting thus in superior crop genotypes. The polygenic basis of heat tolerance and the issues of detecting minor QTLs with molecular markers strongly limit the use of MAS to identify heat tolerance related traits by classical genetics. High throughput sequencing and the predicted decline in genotyping costs will assist in obtaining a denser genome-wide marker coverage for all crop species, accelerate QTL discovery and transfer by GS and confer an increased flexibility to the interplay between phenotypic evaluation and selection (Heffner et al., 2009).

Heat stress tolerance is a polygenic character often measured using complex traits such as yield under stress, which implicate many processes and mechanisms. Therefore, introgression of a gene or QTL by conventional or modern breeding is usually not sufficient to develop heat-tolerant lines, unless there is a large effect on a particular key process. In addition, achieving highest genetic gain via classical methods requires a just choice for the appropriate breeding strategy (i.e., recurrent or GS).

The manipulation of major regulatory genes through biotechnology is considered to be more efficient than conventional breeding through serial hybridizations. Linking several beneficial genes (transgenic pyramiding) into a commercial variety via the transgenic approach is also likely to provide a key route to crop improvement. A likely drawback is that the function of homologous genes is often diverged between species. Thus, for practical reasons, marker-assisted backcrossing of a transgene into a commercially viable variety will be necessary. In addition, to avoid negative effects on plant growth, it will be necessary to restrict transgene expression to a specific tissue or stress, via the use of tissue-specific and stress-inducible promoters. As yet, a limited number of reports have been published on QTL validation or introgression and the field-testing of transgenic plants (Varshney et al., 2011).

Until now, very few tolerant varieties have been released, mainly due to regulatory issues associated with GMOs or due to the difficult and lengthy process of breeding for tolerance. There is also a great concern that laboratory testing does not reflect true field conditions. In many laboratory studies on stress tolerance of genetically modified plants, tolerance is tested either against a single stress or is examined over relatively short periods of time. In contrast, in field conditions, plants are simultaneously subjected to various stresses and in some circumstances these constraints extend throughout their lifetimes. As consequence, to identify and understand these interactions it will be necessary to perform thermotolerance phenotyping with multiple stresses.

Abiotic stresses affect plants in a complex manner depending on the growth stage. The conventional and molecular plant breeding efforts are limited by the present lack of accurate phenotyping for stress responses, due to the incomplete understanding of tolerance mechanisms. For example, developing countries are still lacking in appropriate phenotyping facilities in the public research sector.

In addition, generating high yielding and stress-tolerant crops requires a thorough understanding of the metabolic and developmental processes involved not only in stress responses but also in energy regulation (Hirayama and Shinozaki, 2010). For instance, combining different approaches led to the development of rice cultivars referred to as “Green Super Rice,” tolerant to several stresses and having a high nutritious value, promising to greatly reduce the consumption of pesticides, chemical fertilizers, or water (Yang and Zhang, 2010). Such strategies are likely to improve stress tolerance but nonetheless, there is a lag from basic research to the production of stress-tolerant crops.

Significant emphasis has been put on molecular biology as the newest agricultural research tool to detect molecular markers associated with stress related traits, as well as individual candidate genes for stress tolerance (Barnabás et al., 2008). Nevertheless, its value in providing short-term solutions for heat stress tolerance is unknown due to the complexity of the genetic background for desirable physiological traits and their interaction with the environment (Savin and Slafer, 2010). The use of mathematical modeling in conjunction with genetic information is emerging as an additional methodology to assist the identification of physiological traits for new plant ideotypes (Semenov and Halford, 2009).

Ultimately, differences in their primary concerns and research strategies cause molecular biologists, plant physiologists, and breeders to work independently, where an intimate collaboration amongst them is required. Such integration will enable identification of tolerance genes and elucidate the functional relationships between genotype and observed phenotypes, providing thus a system-wide phenome to genome analysis, while enabling accurate trait mapping, introgression of superior alleles or cloning of major QTLs for complex characters such as abiotic stress tolerance. The transgenic approach must be combined with the efforts in marker-assisted breeding programs for stress related genes and QTLs for obtaining effective high temperature tolerance.

Particularly in the view of the predicted global warming, understanding the molecular basis of the relevant agronomic traits is essential to allow breeders to design new ideotypes *in silico*, construct new genotypes *in planta* and ultimately implement these across a wide range of environmental conditions and locations.

## Conflict of Interest Statement

The authors declare that the research was conducted in the absence of any commercial or financial relationships that could be construed as a potential conflict of interest.
